# Mutant TP53 modulates metastasis of triple negative breast cancer through adenosine A2b receptor signaling

**DOI:** 10.18632/oncotarget.26177

**Published:** 2018-10-02

**Authors:** Eisuke Horigome, Michiru Fujieda, Tadashi Handa, Ayaka Katayama, Masashi Ito, Ami Ichihara, Daiki Tanaka, Navchaa Gombodorj, Shinji Yoshiyama, Arito Yamane, Keiichi Yamada, Jun Horiguchi, Kazuo Shinozuka, Tetsunari Oyama, Masahiko Nishiyama, Susumu Rokudai

**Affiliations:** ^1^ Department of Molecular Pharmacology and Oncology, Gunma University, Gunma, Japan; ^2^ Department of Diagnostic Pathology, Gunma University, Gunma, Japan; ^3^ Division of Molecular Science, Gunma University, Gunma, Japan; ^4^ Department of Breast Surgery, Graduate School of Medical Sciences, International University of Health and Welfare, Chiba, Japan

**Keywords:** ADORA2B, TP53, NF-kB, breast cancer, synthetic lethality

## Abstract

**Purpose:**

The identification of genes with synthetic lethality in the context of mutant TP53 is a promising strategy for the treatment of basal-like triple negative breast cancer (TNBC). This study investigated regulators of mutant TP53 (R248Q) in basal-like TNBC and their impact on tumorigenesis.

**Experimental Design:**

TNBC cells were analyzed by RNA-seq, and synthetic-lethal shRNA knock-down screening, to identify genes related to the expression of mutant *TP53*. A tissue microarray of 232 breast cancer samples, that included 66 TNBC cases, was used to assess clinicopathological correlates of tumor protein expression. Functional assays were performed *in vitro* and *in vivo* to assess the role of ADORA2B in TNBC.

**Results:**

Transcriptome profiling identified ADORA2B as up-regulated in basal-like TNBC cell lines with R248Q-mutated TP53, with shRNA-screening suggesting the potential for a synthetic-lethal interaction between these genes. In clinical samples, ADORA2B was highly expressed in 39.4% (26/66) of TNBC patients. ADORA2B-expression was significantly correlated with ER (*P* < 0.01), PgR (*P* = 0.027), EGFR (*P* < 0.01), and tumor size (*P* = 0.037), and was an independent prognostic factor for outcome (*P* = 0.036). In line with this, ADORA2B-transduced TNBC cells showed increased tumorigenesis, and ADORA2B knockdown, along with mutant p53 knockdown, decreased metastasis both *in vitro* and *in vivo*. Notably, the cytotoxic cyclic peptide SA-I suppressed ADORA2B expression and tumorigenesis in TNBC cell lines.

**Conclusions:**

ADORA2B expression increases the oncogenic potential of basal-like TNBC and is an independent factor for poor outcome. These data suggest that ADORA2B could serve as a prognostic biomarker and a potential therapeutic target for basal-like TNBC.

## INTRODUCTION

TNBC is clinically aggressive and difficult to treat. One of the reasons for the failure to develop new therapies for this subgroup of breast cancer patients has been the difficulty in identifying highly prevalent, targetable molecular alterations in these tumors [1]. The validation of a targeted therapy approach for patients with TNBC is thus urgently needed [2]. The identification of genes that have synthetic lethality with mutant TP53 is a promising approach in this regard, since TP53 mutations occur in approximately 40% percent of all breast cancers, with the highest frequency found in the basal-like (80%) and HER-2-enriched (72%) subtypes of TNBC, and the lowest found in the Luminal A (12%) and Luminal B (29%) subtypes [3, 4]. Breast cancers carrying mutations in TP53 are characterized by an aggressive and metastatic phenotype with the poorest outcomes [5]. Mutations at five known hot-spots in TP53 (V143A, G245S, R248Q, R249S and R273H) are frequently associated with gain-of-function (GOF) phenotypes [6], although it is the loss of p53 function that is more generally considered to play an essential role in carcinogenesis and disease progression [7, 8].

The adenosine A2b receptor (ADORA2B) consists of four members (A1, A2A, A2B, and A3) that belong to the G protein-coupled adenosine receptor superfamily [9]. ADORA2B is mainly localized to the cell membrane and is associated with inflammation and immune responses [10]. Additionally, ADORA2B is selectively up-regulated under hypoxic conditions, and antagonists to this receptor effectively neutralize ATP-mediated changes in post-hypoxic permeability [11]. In this report, we identified ADORA2B as a biomarker of basal-like TNBC using whole transcriptome analysis, and subsequently investigated the association between the expression of ADORA2B and TNBC biology. Based on our findings, we hypothesize that dysregulation of ADORA2B is involved in the progression of basal-like TNBC via an interaction with the GOF phenotype of TP53 hot spot mutations, leading to disease progression and metastasis. These finding suggest that ADORA2B and adenosine signaling play critical roles in tumor development and invasiveness in TNBC, and may represent therapeutic biomarkers for this disease.

## RESULTS

### Identification of genes with synthetic lethality in the context of mutant *TP53*

**Figure 1:**
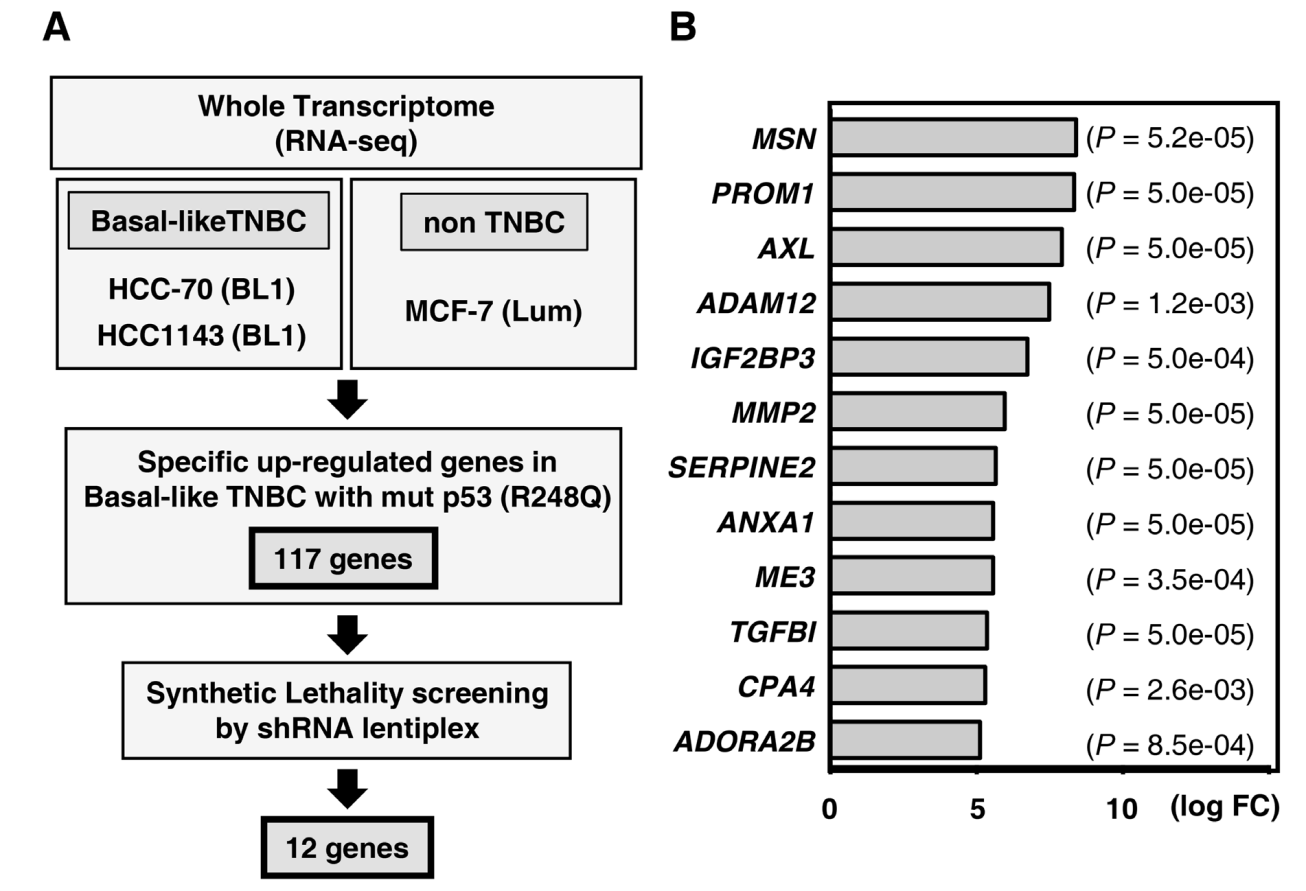
Gene expression profiling to identify synthetic lethal genes in *TP53*-mutated TNBC (**A**) Schematic of the method used to identify synthetic lethal genes in *TP53*-mutated (R248Q) cell lines. A total of 117 significantly up-regulated genes (*P* < 0.05 and FDR < 0.5) were identified by comparison with mutant *TP53*-R248Q cells (HCC-70 and HCC-1143) and wild-type *TP53* cells (MCF-7). (**B**) Subsequently, 12 synthetic lethal candidate genes were identified by shRNA lentiplex screening. The genes are listed in descending order in regards to the relative fold-change in the level of mutant *TP53* (R248Q).

While TNBC can be sub-categorized into six subtypes with distinct characteristics using public gene expression databases, namely basal-like1, basal-like2, immunomodulatory, mesenchymal-like, mesenchymal stem-like, and the luminal androgen receptor (LAR) subtype [12], *TP53* mutations occur in approximately 80 percent of the basal-like subtypes [3, 4]. To identify a therapeutic target for patients with TNBC, we focused on basal-like cases carrying the R248Q TP53 mutation, which is specifically expressed in TNBC, and initially performed a pre-screening whole transcriptome analysis (RNA-seq) to identify important genes. For this we used two basal-like TNBC cell lines carrying R248Q mutated TP53 (HCC-70 and HCC1143) and compared their profiles to that of a non-TNBC cell line carrying wild-type TP53 (MCF-7) (Figure 1A). After alignment of a total of 15,346 genes to the reference sequence, hierarchical cluster analysis identified a total of 171 genes that were significantly up-regulated (FDR-adjusted *p*-values < 0.05) in the basal-like TNBC cells compared to the non-TNBC cells, suggesting a role in the biology of basal-like TNBC carrying R248Q-mutated TP53. To address this possibility in more detail, we carried out Ingenuity Pathway Analysis (IPA), which revealed that more than 30% of the affected genes belonged to the functional classes of “cell signaling”, “antimicrobial response”, and “inflammatory response” (Supplementary Figure 1).

Targeted therapy, in the form of synthetic lethal interactions, is a potentially powerful strategy for the treatment of TP53 mutant tumors. Using a comprehensive shRNA-lentiplex knock-down system (Mission shRNA, Sigma-Aldrich), we thus screened the 171 genes upregulated in basal-like TNBCs for synthetic lethality interactions with mutant TP53. Among the differentially expressed genes (those with > 2 shRNAs), we identified a total of 12 genes that demonstrated synthetic lethality in basal-like TNBC cell lines (Figure 1B and Supplementary Figure 2). Among these candidates, ADORA2B is a G protein-coupled adenosine receptor protein known to be involved in inflammation and immune responses [10]. IPA revealed that *ADORA2B* expression was correlated with tumor survival pathways, including the NF-kB and HIF-1 pathways (Supplementary Figure 3), indicating it may have a role to play in tumorigenicity and metastasis, although such an association has not been previously explored in TNBC.

### Immunohistochemical findings in regard to ADORA2B expression

**Figure 2:**
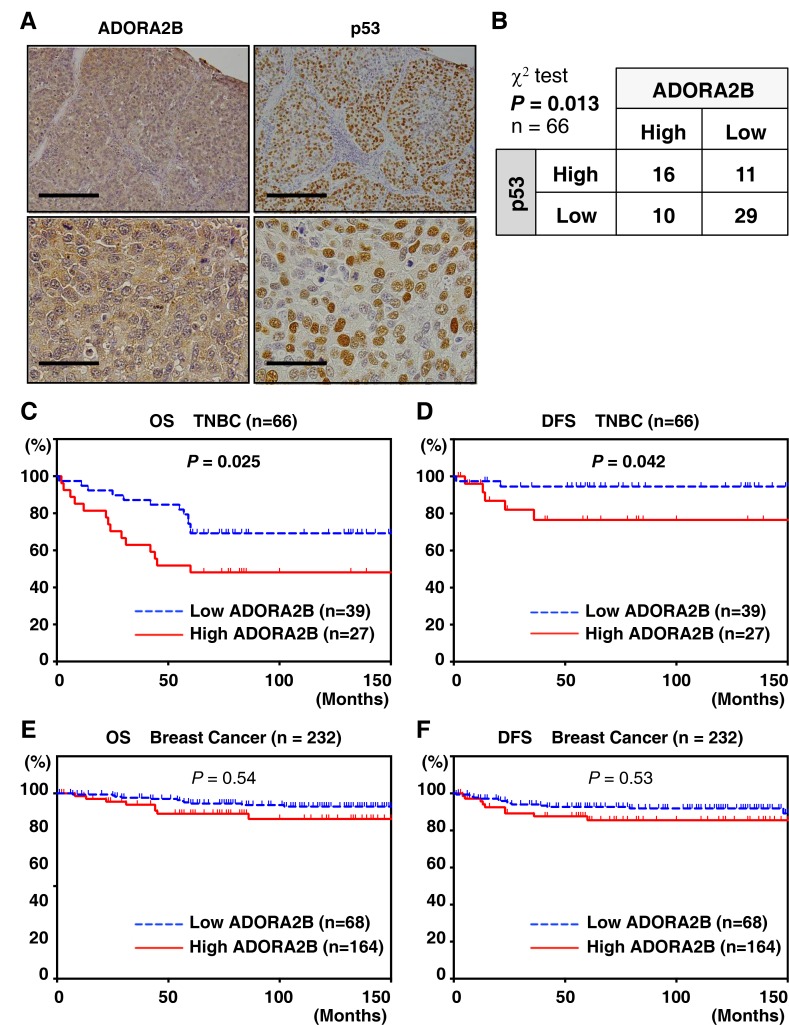
ADORA2B expression is correlated with p53 expression and poor prognosis in TNBC samples (**A**) Representative immunohistochemical staining of a breast cancer sample. ADORA2B immunostaining demonstrates a nuclear and cytoplasmic pattern, with a score of 5. Scale bars are 200 μm. (**B**) Stratification of TNBC patient samples (*n =* 66) based on the expression of ADORA2B and p53. The chi-square test was performed for the association (*P* = 0.013). (**C**, **D**) Kaplan–Meier analysis of survival in TNBC patients grouped by ADORA2B expression (*n* = 66). A statistically significant difference in OS and PFS was observed between high ADORA2B expression patients and those with low ADORA2B expression (OS, *P* = 0.025; PFS, *P* = 0.042). (**E**, **F**) Kaplan–Meier analysis of survival in breast cancer patients grouped by ADORA2B expression (*n* = 232). No statistically significant difference in OS and PFS was observed between those with high or low ADORA2B expression (OS, *P* = 0.54; PFS, *P* = 0.53).

Based on this observation, the expression of ADORA2B was examined in clinical samples of TNBC using a tissue microarray. All 232 TNBC patients in this study had evidence of breast cancer and received radical surgery, including the 66 TNBC cases (Tables 1 and 2). ADORA2B was highly expressed in breast cancer patients in general, as well as in TNBC patients (29.3% (68/232), and 39.4% (26/66), respectively). Clinicopathological analysis showed that high ADORA2B expression has significantly reverse correlation with four important clinical parameters, ER positivity (*P* < 0.01), PgR positivity (*P* = 0.027), EGFR positivity (*P* < 0.01) and tumor size (*P* = 0.037), but not HER-2 positivity (*P* = 0.77) (Table 1). Representative examples of ADORA2B and p53 protein expression in tumor tissue samples are shown in Figure 2A. While ADORA2B was not observed in non-cancerous tissues, elevated ADORA2B expression was observed in both the nucleus and cytoplasm of malignant cells.

**Table 1:** Patient’s demographics according to ADORA2B expression

	ADORA2B expression					ADORA2b_TNBC				
Variables	High (*n* = 68)		Low (*n* = 164)			High (*n* = 26)		Low (*n* = 40)		
	No.	(%)	No.	(%)	*P*−value	No.	(%)	No.	(%)	*P*−value
Age					0.086					<0.01
>65 yr	22	(9.5)	31	(13.4)		12	(18.2)	6	(9.1)	
≤65yr	46	(19.8)	133	(57.3)		14	(21.2)	34	(51.5)	
Subtype					0.092					−
Luminal A	27	(11.6)	91	(39.2)		−		−		
Luminal B	3	(1.3)	7	(3.0)		−		−		
HER2	12	(5.2)	26	(11.2)		−		−		
TNBC	26	(11.2)	40	(17.3)		−		−		
ER					<0.01^*^					−
Positive	28	(12.1)	98	(42.2)		−		−		
Negative	40	(17.2)	66	(28.4)		−		−		
PgR					0.027^*^					−
Positive	20	(8.6)	74	(31.9)		−		−		
Negative	48	(20.7)	90	(38.8)		−		−		
HER2					0.77					−
Score 0, 1+	54	(23.3)	133	(57.3)		−		−		
Score 2+, 3+	14	(6.0)	31	(13.4)		−		−		
EGFR					<0.01^*^					0.32
Score 0, 1+	56	(24.1)	154	(66.4)		18	(27.3)	32	(48.5)	
Score 2+, 3+	12	(5.2)	10	(4.3)		8	(12.1)	8	(12.1)	
CK5/6					0.22					0.74
Positive	5	(2.2)	6	(2.6)		4	(6.1)	5	(7.6)	
Negative	63	(27.1)	158	(68.1)		22	(33.3)	35	(53.0)	
Ki67 labeling index										
	22.3 ± 25.9		18.8 ± 21.6			36.3 ± 31.7		38.2 ± 30.1		
T factor					0.045^*^					0.31
T1	25	(10.8)	84	(36.2)		11	(16.7)	22	(33.3)	
T2-4	43	(18.5)	80	(34.5)		15	(22.7)	18	(27.3)	
N factor					0.21					0.93
N0	45	(19.4)	94	(40.5)		14	(21.2)	22	(33.3)	
N1-2	23	(9.9)	70	(30.2)		12	(18.2)	18	(27.3)	
M factor					0.15					>0.99
M0	66	(28.4)	163	(70.3)		26	(39.4)	40	(60.6)	
M1	2	(0.9)	1	(0.4)		0	(0.0)	0	(0.0)	
Disease stage					0.69					0.33
I	23	(9.9)	60	(25.9)		8	(12.1)	17	(25.8)	
II-IV	45	(19.4)	104	(44.8)		18	(27.3)	23	(34.8)	
Lymphatic permeation					0.97					0.37
Positive	47	(20.2)	113	(48.7)		19	(28.8)	25	(37.9)	
Negative	21	(9.1)	51	(22.0)		7	(10.6)	15	(22.7)	
Venous invasion					0.78					0.78
Positive	22	(9.5)	50	(21.6)		10	(15.2)	14	(21.2)	
Negative	46	(19.8)	114	(49.1)		16	(24.2)	26	(39.4)	
Nuclear grade					0.10					0.010^*^
NG1	9	(3.9)	37	(15.9)		2	(3.0)	4	(6.1)	
NG2	18	(7.7)	44	(19.0)		2	(3.0)	4	(6.1)	
NG3	41	(17.7)	83	(35.8)		22	(33.3)	32	(48.5)	

^*^P-values were obtained by Fisher’s exact test.

**Table 2:** Univariate and multivariate survival analysis in TNBC patients

Variables	Overall survival				Progression-free survival			
	Univariate		Multivariate		Univariate		Multivariate	
	5-yrs rate (%)	*P*-value	HR (95% CI)	*p*-value	5-yrs rate (%)	*P*-value	HR (95% CI)	*P*-value
Age		0.015		0.18		0.78		
≤ 65yr	68.7		1.88		88.9			
> 65 yr	38.9		(0.75–4.73)		83.6			
T factor		0.83				0.19		
T1	60.7				88.2			
T2-3	60.6				82.4			
N factor		0.44				0.029^*^		0.018^*^
N0	55.5				93.2		**8.11**	
N1-2	66.7				78.4		**(1.38–153.4)**	
Disease Stage		0.41				0.16		
I	52.0				95.4			
II–III	65.9				83.4			
Lymphatic permeation		0.8				0.36		
Positive	61.4				78.6			
Negative	59.1				90			
Vascular invasion		0.59				0.96		
Positive	58.3				85.6			
Negative	61.9				81.4			
ADORA2B expression		0.025^*^		0.24		0.042^*^		0.036^*^
High expression	46.2		1.74		75.4		**5.18**	
Low expression	70.0		(0.69–4.31)		94.7		**(1.11–36.3)**	

To address the clinical significance of ADORA2B expression, we investigated whether high expression of this protein correlates with p53 expression. We found that among the 66 TNBC patients, those with higher p53 levels of expression also tended to have higher levels of ADORA2B, and *vice versa* (chi-square test, *P* = 0.013; Figure 2B). To evaluate the significance of ADORA2B expression for survival, we then assessed postoperative OS and PFS by Kaplan–Meier analysis. ADORA2B-positive patients showed poor OS (log-rank *P* < 0.01) and PFS (log-rank *P* < 0.01) compared to those who were ADORA2B-negative (Figure 2C and 2D). The five-year survival rates and median survival times for the entire breast cancer cohort were 50.2% and 38.3 months (0.75 to 111.5 months), respectively (Figure 2E and 2F). These results indicate that ADORA2B could be a biomarker for predicting poor outcome in some TNBC patients, but not in all breast cancer cases.

### The GOF mutant *TP53* (R248Q) induces *ADORA2B* expression

**Figure 3:**
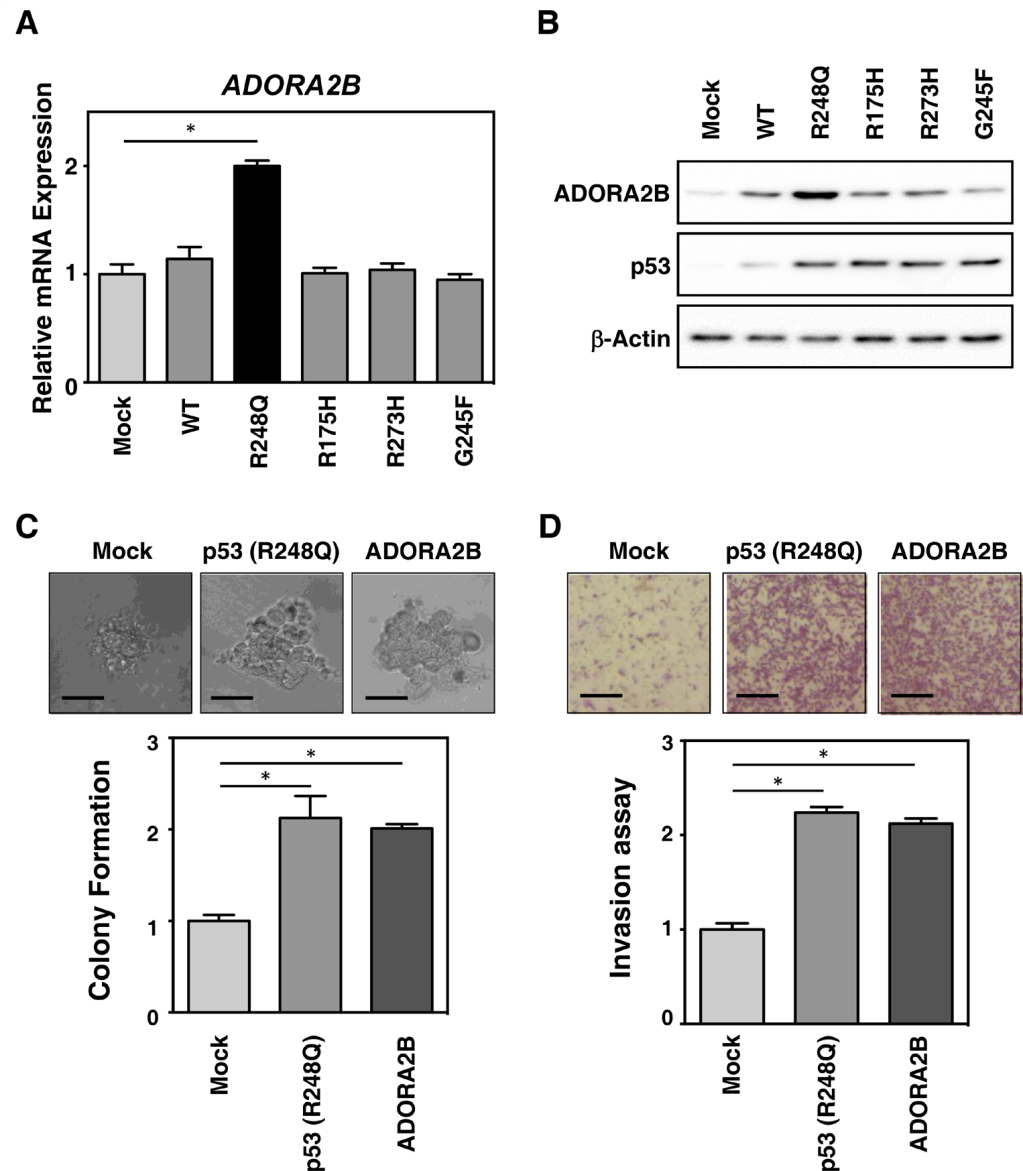
Mutant *TP53* (R248Q) induces *ADORA2B* expression and tumorigenesis in breast cancer cells (**A**) MCF-7 cells were retrovirally transduced with empty vector control (Mock), or plasmids expressing wild-type or mutant *TP53* (R248Q, R175H, R273H and G245F). The mRNA levels of *ADORA2B* were subsequently determined by real-time RT-PCR. (**B**) The cells transduced as in (A), were subjected to immunoblotting using antibodies against ADORA2B, p53, and β-actin (as a loading control). (**C**) Breast cancer tumorigenesis is increased by mutant *TP53* (R248Q) expression. HCC70 cells were retrovirally transduced with empty vector control (Mock), mutant *TP53* (R248Q), or *ADORA2B* expression plasmids. Anchorage-independent growth of the cells after transduction was monitored by soft agar colony formation assay. (**D**) Breast cancer metastasis is increased by mutant *TP53* (R248Q). The cells transduced as in (C), were evaluated by invasion assay. Data in bar graphs represent mean ± SD; ^*^P < 0.05.

The adenosine A2b receptor (ADORA2B) belongs to the G protein-coupled adenosine receptor superfamily, and is expressed in the plasma membrane [9, 10], suggesting it may play a role in interacting with the tumor microenvironment (TME). To confirm the enhanced expression of ADORA2B in mutant TP53-expressing tumors, the MCF-7 cell line was transduced with either wild-type or mutant *TP53* (R248Q, R175H, R273H, and G245F) using retroviruses. As shown in Figure 3A and 3B, several mutants of *TP53*, especially R248Q, resulted in clones expressing high levels of ADORA2B mRNA (Figure 3A) and protein (Figure 3B), consistent with our finding in 66 resected TNBC samples. The results indicate that R248Q-mutated TP53 regulates ADORA2B expression in breast cancer cells.

To further evaluate the functional relevance of ADORA2B and mutant TP53 expression during tumor progression, we monitored the colony formation of HCC-70 cells retrovirally transduced with wild-type and mutant TP53 (R248Q, R175H, R273H, and G245F). As shown in Figure 3C and Supplementary Figure 4, ADORA2B and mutant TP53 transduction in breast cancer cells, led to increased anchorage-independent colony formation in soft agar. Consistent with this, ADORA2B and R248Q-*TP53* induction also increased invasive potential (Figure 3D). These results indicate that R248Q-mutated *TP53* regulates ADORA2B expression in TNBC, and that ADORA2B may serve as a novel TNBC biomarker for prognosis.

### ADORA2B-depletion represses TNBC tumor growth and metastasis *in vitro*

**Figure 4:**
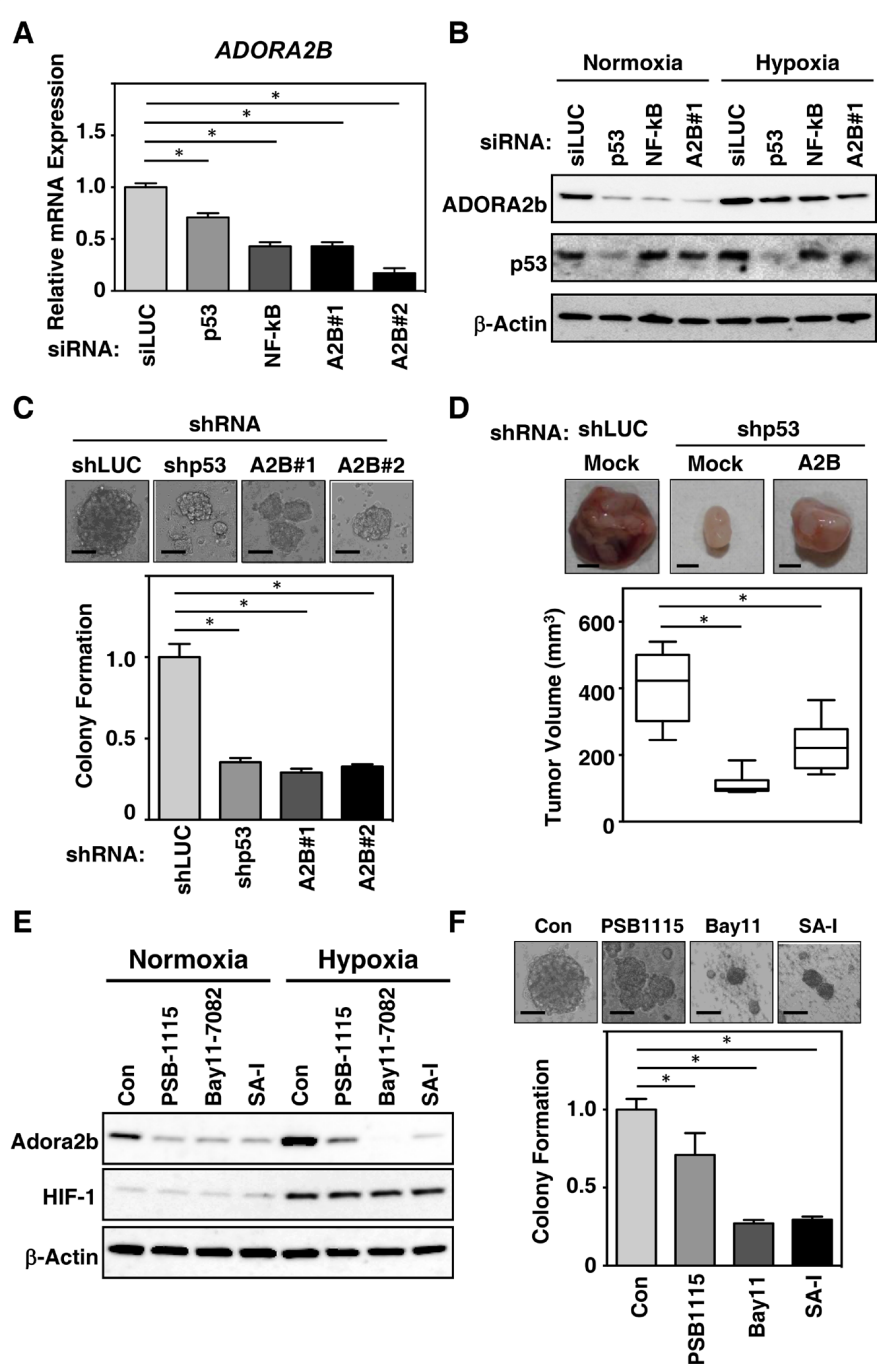
Mutant *TP53* depletion inhibits breast cancer tumorigenesis and modulates *ADORA2B* induction through a gain-of function of mutant *TP53* (**A**) The breast cancer cell line HCC-70 was treated with siRNAs for either luciferase (siLUC) as a control, TP53, NF-kB or ADORA2B (A2B#1 and A2B#2). Induction of *ADORA2B* expression was determined by real-time RT-PCR. (**B**) HCC-70 cells were treated with siRNAs as in (A), and cultured under either normoxic or hypoxic condition for 24 h. Cell lysates were subsequently analyzed by immunoblotting analysis using anti-ADORA2B, TP53, or β-actin antibodies. (**C**) Breast cancer tumorigenesis is decreased by the shRNA-mediated depletion of mutant *TP53*. HCC-70 cells were treated with shRNAs for either luciferase (siLUC) as a control, TP53, or ADORA2B. Anchorage-independent growth after depletion was monitored by soft agar colony formation. (**D**) p53-depletion induces tumorigenesis through ADORA2B in breast cancer cells. The growth of p53-depleted HCC-70 cells after induction of ADORA2B was monitored by xenograft assay. The growth of ADORA2B-expressing HCC-70 cells after depletion of luciferase as a control (shLUC) or mutant p53, was monitored by xenograft assays. Representative images of xenografts from subcutaneously transplanted are shown. The results of six independent injections of knockdown cells are shown. Twenty days after implantation, the length (L) and width (W) of the tumor mass were measured, and the tumor volume (TV) was calculated using the equation: TV = (L × W2)/2. ^*^P < 0.05. (**E**) The cyclic peptide SA-I decreases breast cancer tumorigenesis via ADORA2B. HCC-70 cells were treated with 10 μM of PSB-1115, 10 μM of Bay 11-7082, or 10 μM of cyclic peptide SA-I for 24 h. Cell lysates were subsequently immunoblotted with anti-ADORA2B, HIF1, or β-actin antibodies. (**F**) HCC-70 cells treated with 10 uM of PSB-1115, 10 uM of Bat11-7082, or 10 uM of SA-I were monitored for growth by soft agar colony formation assay. Data in bar graphs represent mean ± SD; ^*^P < 0.05.

We next examined the oncogenic role of p53 and NF-kB in regulating the expression of ADORA2B in basal-like TNBC cell lines, using a loss-of-function approach. siRNAs against TP53, NF-kB and ADORA2B were transfected into the HCC-70 cell line, with an siRNA targeting luciferase (siLUC) used as a control. TP53 and NF-kB knockdown cells showed low expression of ADORA2B mRNA (Figure 4A) and protein (Figure 4B), consistent with our findings in resected patient TNBC samples. Subsequently, we investigated the impact of hypoxia on cancer progression and metastasis. The higher expression of ADORA2B under hypoxic conditions in HCC-70 cells, compared with normoxic conditions (Figure 4B), is consistent with the idea that ADORA2B may be upregulated in the hypoxic TME.

In order to evaluate the functional relevance of mutant TP53 and *ADORA2B* expression during tumor formation, we next investigated the colony formation of HCC-70 cells lentivirally transduced with TP53 or ADORA2B shRNAs. As shown in Figure 4C, TP53 or ADORA2B knockdown in HCC-70 cells, led to decreased anchorage-independent colony formation in soft agar. Subcutaneous transplantation of TP53-knockdown clones into immunodeficient mice resulted in suppressed tumor formation compared with control luciferase shRNA xenografts (Figure 4D, top left and top middle panels). ADORA2B knockdown also suppressed tumor growth (Figure 4D, bar graph). However, knockdown of ADORA2B in TP53-supressed cells, resulted in larger tumor growth than in tumors with TP53-supression alone (Figure 4D, top right panel). Together, these data suggest that downregulation of TP53 decreases ADORA2B expression and suppresses tumor formation.

### ADORA2B is a potential therapeutic target in TNBC

Finally, we investigated the sensitivity of ADORA2B-expressing cells to specific inhibitors for ADORA2B (PSB-1115) and NF-kB (Bay 11-7082). Interestingly, the cyclic peptide originally designed for growth inhibition in breast cancer [13, 14] showed the biggest suppression of ADORA2B, especially under hypoxic conditions, and reduced anchorage-independent colony formation to levels equal to, or lower than, those observed with the use of PSB-1115 and Bay 11-7082 (Figure 4E and 4F). This suggests that a new molecular sub-classification of TNBC is required, especially among cases with basal-like phenotypes, with tumor growth being driven by an interaction of ADORA2B-signaling with the TME. In summary, our results indicate that ADORA2B has oncogenic activity both *in vitro* and *in vivo*, and suggest that this receptor could be a critical driver of tumor propagation through the adenosine signaling pathway. This raises the possibility that this pathway may represent a novel therapeutic target in TNBC.

## DISCUSSION

Despite recent advances in the targeted therapy of breast cancer based on patterns of ER, PgR, and HER2 receptor expression, which have resulted in the extension of survival rates, the identification and validation of a targeted therapy for patients with TNBC is currently one of the most urgent needs in breast cancer therapeutics [12]. In this study, we report, for the first time, that *ADORA2B* expression is frequent in TP53-mutated basal-like TNBC samples. Importantly, we have demonstrated that ADORA2B may play a critical role in the progression (proliferation and metastasis) of breast cancer.

Growing evidence indicates that the adenosine-receptor pathway may be a promising therapeutic target in a wide range of conditions, including cerebral and cardiac ischemic diseases, sleep disorders, immune and inflammatory disorders, and cancer [15, 16]. ADORA2B requires higher concentrations of adenosine for activation (stimulation of adenylate cyclase and phospholipase C) than the A1, A2A and A3 subtypes [9]. This indicates that ADORA2B may play an important role in pathophysiological conditions that are associated with massive adenosine release, especially in the TME [17]. In the ischemic TME of solid tumors, hypoxia-inducible factor 1 alpha (HIF-1α) is activated by signal transduction pathways involving extracellular regulated kinase 1/2 (ERK1/2) and Akt [18]. Excessive adenosine production in ischemic solid tumors, stimulated by HIF-1α, is transported to the TME by equilibrative nucleoside transporters (ENTs), which are found in the plasma membrane of most cells [19, 20].

It has been suggested that targeting mutant forms of p53 protein, or its regulators, might be an effective approach for the treatment of TNBC [17]. Many cancers express mutant p53 proteins that have lost wild-type tumor suppressor activity and/or have acquired additional oncogenic functions that contribute to tumor progression. Indeed, mutation of *TP53* defines a key step in the progression towards aggressive and metastatic breast cancer with the poorest outcome [5]. The structural changes introduced by five hot-spot mutations (V143A, G245S, R248Q, R249S, and R273H) in *TP53* have been evaluated by chemical-shift changes. Mutants of p53 that affect protein conformation (such as R175H) show strong binding to p63 and p73, whereas p53 mutants that only mildly affect protein conformation (such as R273H) bind less well [21, 22]. The sequestering of p63 by mutant p53 has been linked with metastatic risk in breast cancer patients [23, 24]. On the other hand, the *TP53* hot spot mutation R248Q has been associated with an oncogenic GOF phenotype in breast cancer [25]. Our data reveal that this *TP53* mutant can induce the expression of *ADORA2B*, something that could be mediated by CCAAT box and NF-Y protein interactions. This would be consistent with another study showing that mutant p53 can interact with NF-Y transcription factors to stimulate the expression of proliferative cell cycle genes after DNA damage [26]. In summary, the findings from this study are consistent with ADORA2B activity being important for tumor progression in at least a subset of breast cancer, providing a potential therapeutic target for the development of small molecule inhibitors for the treatment of this devastating disease.

## MATERIALS AND METHODS

### Cell culture

The human breast cancer cell lines, MCF-7, HCC70, and HCC1143, and the HEK293T cell line, were obtained from the RIKEN Cell Bank (Tsukuba, Japan) and the American Type Culture Collection (ATCC, Manassas, VA, USA). Cell culture was previously described [27]. In short, the cells were cultured in RPMI 1640 medium supplemented with 100 U/mL penicillin, 100 U/mL streptomycin, and 10% fetal bovine serum in a humidified atmosphere, at 37° C in a 5% CO_2_ incubator. For hypoxic induction, the cells were exposed to hypoxia (1% O_2_) using the hypoxic chamber (Billups-Rothenberg, Del Mar, CA, USA). The inhibitors for ADORA2B (PSB-1115) and NF-kB (Bay 11-7082) were purchased from Sigma-Aldrich (St Louis, MO). SA-I is previously described [13, 14].

### Patients

We retrospectively analyzed tumor specimens from 232 patients with primary breast cancer, who underwent surgery for excision of a primary tumor between January 1999 and October 2010 at Gunma University Hospital. The median age of the patients was 72 years (range 56–84). All patients received radical surgery, with evidence of pathological stage I in 35.8% (83/232), stage II in 44.4% (103/232), stage III in 15.1% (35/232), and stage IV in 4.7% (11/232) of patients. Lymphatic permeation and venous invasion were observed in 69.0% (160/232) and 31.0% (72/232) of patients, respectively. Tumor staging was based on the Union for International Cancer Control (UICC) TNM classification criteria, seventh edition [28]. Nuclear grades were defined using the sum of the scores for nuclear atypia (1 = low-degree atypia; 2 = intermediate-degree atypia; 3 = high-degree atypia) and mitotic count (mitoses per 10 high-power fields, × 40 objective lens: 1 = 0–5 mitoses; 2 = 6–12 mitoses; 3 = ≥ 13 mitoses). The nuclear grade was defined as 1, 2, or 3, when the combined scores for nuclear atypia and mitoses were 2–3, 4, or 5–6, respectively. This research conformed to the Declaration of Helsinki and the guidelines of the Gunma University Ethics Review Board for Medical Research Involving Human Subjects (Approval Number: 150044).

### Tissue microarrays (TMAs) and immunohistochemistry

Clinical formalin-fixed paraffin-embedded (FFPE) samples were stored in the archives of the Clinical Department of Pathology, Gunma University Hospital, as previously described [29]. For each patient, one paraffin block containing representative areas of non-necrotic tumor was selected. Breast cancer tissue cores (2.0-mm diameter per tumor) were punched out from representative areas near the invasive front and transferred to the recipient paraffin block in duplicate using a tissue array instrument (Beecher Instruments, Silver Spring, MD).

Immunohistochemical analysis was performed on FFPE sections as previously described [30]. In brief, the sections were de-paraffinized, blocked in 0.25% Casein/ 1% BSA for 30 min, and incubated overnight with diluted primary antibodies at 4° C in a humidified chamber. Staining reactions were developed using the Histofine Simple Stain MAX-PO (Multi) Kit (Nichirei, Tokyo, Japan), with Meyer’s hematoxylin (IHC world, Woodstock, MD, USA) used as a nuclear counterstain. Protein expression was determined using mouse monoclonal antibodies for ADORA2B (1:100 dilution; Abcam, Cambridge, MA, USA) and p53 (DO7, 1:50 dilution; Novocastra/Leica Biosystems, Wetzlar, Germany). ADORA2B expression was scored using a semi-quantitative method, based on the proportion of cells with positive nuclear staining: 1 ≤ 10%, 2 = 10–25%, 3 = 25–50%, 4 = 51–75%, and 5 = ≥ 75% of cells. Tumors that were scored as 4 or 5 using this method were defined as being ADORA2B-positive; those with scores of < 4 were defined as being ADORA2B-negative. Areas of tumors with high cellularity were evaluated for Ki-67 expression, with nuclear staining of any intensity being defined as positive expression. Approximately 1,000 nuclei were counted on each slide, and the proliferative activity was assessed as the percentage of Ki-67-stained nuclei (Ki-67 labeling index) in the sample. Tumors with greater than the median value of the Ki-67 labeling index were defined as being highly proliferative. Sections were assessed under light microscopy in a blinded fashion by at least two of the authors.

### Immunohistochemical evaluation and intrinsic subtype determination

Human epidermal growth factor receptor 2 (HER2) expression was scored according to the American Society of Clinical Oncology/College of American Pathologists guidelines (0 = no reactivity, or membranous reactivity in < 10% of cells; 1+ = faint/barely perceptible membranous reactivity in at least 10% of cells, or reactivity in only part of the cell membrane; 2+ = weak to moderate complete membranous reactivity in at least 10% of tumor cells; 3+ = strong complete membranous reactivity in at least 10% of tumor cells). EGFR expression was scored in the same way as HER2, with scores of 0 or 1+ considered negative, and scores of 2+ or 3+ considered positive. The cutoff value for estrogen receptor (ER) and progesterone receptor (PgR) positivity was 10%. We defined breast cancer subtypes as follows: Luminal A-like (ER or PgR+, and HER2 0/1+), Luminal B-like (ER or PgR+, and HER2 2+/3+), HER2-like (ER−, PgR−, and HER2 2+/3+), and TNBC-like (ER−, PgR−, and HER2 0/1+).

### Genome-wide transcriptome analysis and real-time RT-PCR

RNA analysis was previously described [31, 32]. In short, total RNA was prepared from cell lines using the RNeasy Mini kit (Qiagen, Hilden, Germany). The quality of the RNA was assessed by RNA integrity number (RIN) using the Agilent RNA6000 Pico Kit and the Agilent 2100 Bioanalyzer (Agilent Technologies, Santa Clara, CA, USA). Samples used for RNA-seq had an average RIN value of 9.4 and a minimum RIN value of 8.9. Library preparation was performed using the TruSeq RNA Sample Prep Kit v2 (Illumina, San Diego, CA, USA) from 1 µg of total RNA, according to the manufacturer’s protocol. The resulting libraries were subjected to single end sequencing using the NextSeq500 High Output v1 Kit and the Illumina NextSeq 500 system (76-bp reads; Illumina). Data processing and analyses were performed using TopHat and Cufflinks (Illumina). Briefly, reads were filtered, trimmed, and aligned against the UCSC human reference genome 19 (hg19) using a Tophat2 (v2.0.7) and Bowtie1 (0.12.9) pipeline. Assembly of transcripts was performed using Cufflinks 2.1.1, and differentially expressed transcripts detected with Cuffdiff 2.1.1. Genes with a false-discovery rate (FDR)-adjusted *p*-value < 0.05, and a log2 fold change (TNBC/non-TNBC) > 5, were defined as being significantly up-regulated in TNBC cells.

For real-time RT-PCR, relative RNA quantities were measured using the Universal Probe Library set (Roche), with the KAPA Master mix (KAPA Biosystems, Wilmington, MA, USA). The Universal Probe Library Human *ACTB* Gene Assay (Roche) was used as an endogenous normalization control [31, 33]. Sequence amplification was measured using the StepOne (Thermo Fisher Scientific, Waltham, MA) with relative fold induction determined using the comparative threshold cycle method; standard curves were generated by plotting the observed Ct values against the standard dilutions of a positive control sample. In all experiments, the average of three independent reactions is shown, with error bars indicating standard deviation.

### Statistical analysis

Fisher’s exact test was used to examine the association between two categorical variables. Correlation between variables was analyzed using the non-parametric Spearman’s rank test. Follow-up assessments for the 232 patients were made using medical records, with the Kaplan–Meier method used to evaluate survival and differences analyzed by log-rank test. The day of surgery was defined as the starting day for measuring postoperative survival. Overall survival (OS) was determined as the time from tumor resection to death from any cause. Recurrence-free survival (RFS) was defined as the time between tumor resection and the first disease progression or death. Multivariate analyses were performed using a stepwise Cox proportional hazards model to identify independent prognostic factors. Statistical analysis was performed using the JMP software (SAS, Institute Inc., Cary, NC, USA). In all tests, *P* values < 0.05 were considered statistically significant.

### Anchorage-independent growth and Invasion assay

Breast cancer cell lines were transduced with lentiviruses carrying shRNAs for luciferase (LUC), TP53, or ADORA2B. For soft agar colony formation assays, the cells were grown in triplicate for 10 days, after which anchorage-independent growth was quantified with the CytoSelect-96 kit (Cell Biolabs, San Diego, CA, USA) as previously described [31, 32]. For invasion assays, the transduced breast cancer cells were seeded on the collagen membrane and were incubated for 48 hrs, after which invaded cells were measured with CytoSelect Cell Invasion assay (Cell Biolabs).

### Subcutaneous xenografts

A total of 5 × 10^6^ lentivirally transduced or retrovirally expressed cells were injected subcutaneously into nude mice (BALB/c-nu/nu, CLEA Japan, Tokyo, Japan) and tumor size was measured after 20 days (HCC70). All animal procedures were performed with the approval of the Animal Ethics Committee of Gunma University.

## SUPPLEMENTARY MATERIALS FIGURES


