# Supplementary material for: Mutant TP53 modulates metastasis of triple negative breast cancer through adenosine A2b receptor signaling
Source: Oncotarget. 2018 Oct 2;9(77):34554–66. doi: 10.18632/oncotarget.26177 (PMC6195371; doi:10.18632/oncotarget.26177)
Supplement: Supplementary file 1 [file oncotarget-09-34554-s001.pdf]

Mutant TP53 modulates metastasis of triple negative breast cancer through adenosine A2b receptor signaling

SUPPLEMENTARY MATERIALS

ID	Molecules in Network	Score	Focus Molecules	Top Diseases and Functions
1	**ADORA2B**, Akt, APOL1, APOL3, CASP10, CCL5, CD14, Collagen Alpha1, EMP1, FYB, HIC1, IFI6, IFI16, IFIT2, IFIT3, IFN alpha/beta, IFN Beta, Ifnar, IGFBP3, IL12 (complex), Interferon alpha, LCN2, LRAT, MCTP1, N-cor, NOV, Pka, PROM1, RARRES3, RSAD2, SLPI, STAT5a	47	25	Cell Signaling<br>Antimicrobial Response<br>Inflammatory Response
2	Alpha catenin, C3, CASP1, CASP14, caspase, CFB, Cg, CLEC7A, CP, DSG3, FBLN2, HMGA2, Ige, KLK5, KRT17, LAMA3, LAMA4, LAMB3, LAMC2, Ldh (complex), Mek, NFkB (complex), NLRP1, PAPPA, PARP, Pkc(s), PTGS2, Rac, S100A9, SLC1A3, ST8SIA1, TM4SF1, Vegf, WNT5A, XDH	44	24	Ophthalmic Disease<br>Organismal Injury and Abnormalities<br>Connective Tissue Disorders
3	ANXA1, Ap1, Collagen type II, Collagen(s), CXCL1, CXCL8, EPHB1, ERK1/2, estrogen receptor, F3, Fcer1, Fibrinogen, Focal adhesion kinase, Gm-csf, Hsp27, IFIT1, LDL, LOX, LOXL2, Mapk, MME, MMP14, MSN, MUC4, Nfat (family), PDGF BB, PI3K (complex)	25	16	Cardiovascular System Development and Function<br>Cellular Movement<br>Organismal Development
4	26s Proteasome, Alp, CALD1, CD3, Creb, ERK, ETS1, FSH, GBP1, Hdac, HDAC9, Histone h3, Histone h4, Hsp90, IFI44, IFI44L, Iga, IGF2BP3, IgG, Igm, Jnk, KIT, KRT16, Lh, P38 MAPK, RNA polymerase II, SERPINB5, SERPINE2, SNAI2, TCF7, TCR, Tgf beta, TGFBI, TPM2,	25	16	Dermatological Diseases and Conditions<br>Developmental Disorder<br>Hereditary Disorder
5	ACACA, APOL6, C1QTNF1, CALB2, CCDC80, CDO1, CEBPG, CIB2, COL1A1, CP, CR1L, Defb3, DSC3, IFI27, IFNG, LAIR1, LAMC2, LECT2, LIPE, LIPG, NOV, Nr1h, P2RY6, P3H2, PLA2G2D, PLTP, RARRES1, RARRES3, TIMP4, TNF, TNFSF9, Traj18, U1 snRNP, ULBP2, WNT5B	23	15	Lipid Metabolism<br>Small Molecule Biochemistry<br>Cell-To-Cell Signaling and Interaction
6	ADGRE2, CLDN7, CLIP4, CTNNB1, CYP2A6, EDIL3, ESR1, FUT3, FUT9, GLS, GSTP1, GUCY1A3, GUCY1B3, HIF1A, HNF1A, IgG1, KRT5, LOXL2, MAML1, ME3, METTL7A, NNMT, NR1I2, PDE1C, PLEKHG1, PTPRG, RELA, SERPINB7, SYK, TCN2, TEAD2, TGM2, TMEM45A, TMOD2	19	13	Cancer<br>Drug Metabolism<br>Organismal Injury and Abnormalities
7	ADAM12, ALDH1A3, BGN, BHLHE40, CD109, CPA4, DNAH2, DUSP5, ECSIT, ENG, IL1RAP, JAG1, JAG2, KHSRP, KRT10, KRT6A, mir-515, MMP10, MUC2, NOTCH3, OLR1, PLSCR4, S100A2, SKIL, SMAD4, SMAD1/5, Sos, STAT5A, TGFBR1, TGFBR2, TNFRSF18, TP63, TRAF4, TTC22, VILL	13	10	Cancer<br>Organismal Injury and Abnormalities<br>Reproductive System Disease

Supplementary Figure 1: Top disease and functions identified by IPA analysis for the 171 genes differentially expressed in *TP53*-mutated TNBC cell lines (important biological functions relevant to the genes identified).

ID	Upstream Regulator	Molecule type	Activation z-score	*P*-value	Target molecules in dataset
1	**IFNG**	Cytokine	3.90	6.33E-20	**ADORA2B**, ALDH1A3, APOL6, C3, CALB2, CASP1, CCL5, CD14, CLEC7A, CP, CXCL1, CXCL8, GBP1, HDAC9, IFI16, IFI27, IFI44, IFI44L, IFI6, IFIT1, IFIT2, IFIT3, LAMC2, LCN2, LOX, PLAU, PTGS2, RARRES1, RARRES3, RSAD2, SLPI, TRIM22
2	**TNF**	Cytokine	2.13	2.27E-22	**ADORA2B**, ALDH1A3, C3, CASP1, CCDC80, CCL5, CD14, CFB, CP, CXCL1, CXCL8, DSC3, EMP1, ETS1, F3, GBP1, GSTP1, HDAC9, IFI16, IFIT3, IGFBP3, LAMA3, LAMB3, LAMC2, LCN2, LOX, MMP14, NOV, PLAU, PTGS2, RARRES3, RCAN2, S100A9, SERPINE2, TM4SF1, WNT5A
3	**NFkB**	Complex	1.76	1.17E-07	**ADORA2B**, C3, CCL5, CXCL1, CXCL8, F3, KIT, KRT17, LCN2, PLAU, PTGS2, RSAD2, SNAI2
4	**HIF1A**	Transcription Regulator	1.58	8.04E-07	**ADORA2B**, CXCL8, ETS1, IGFBP3, LOX, LOXL2, NOV, PROM1, PTGS2, TLR6, TMEM45A
5	**IL1A**	Cytokine	1.48	1.58E-09	**ADORA2B**, ALDH1A3, CCL5, CXCL1, CXCL8, LCN2, LOX, PLAU, PTGS2, S100A9

Supplementary Figure 2: Causal networks associated with *ADORA2B* expression in BC cells by IPA analysis.

Gene Symbol	Numbers of shRNA	TRCN	MCF-7 (WT)	HCC70 (R248Q)	HCC1143 (R248Q)
ADORA2B	4	TRCN0000065336			
		TRCN0000065334			
		TRCN0000289318			
		TRCN0000065337			
ME3	4	TRCN0000064835			
		TRCN0000064834			
		TRCN0000064836			
		TRCN0000064833			
ADAM12	3	TRCN0000047036			
		TRCN0000047037			
		TRCN0000047034			
ANXA1	3	TRCN0000056100			
		TRCN0000289716			
		TRCN0000056099			
AXL	3	TRCN0000342414			
		TRCN0000000575			
		TRCN0000001040			
CPA4	3	TRCN0000290601			
		TRCN0000046992			
		TRCN0000046990			
IGF2BP3	3	TRCN0000074674			
		TRCN0000072510			
		TRCN0000072509			
MMP2	3	TRCN0000051524			
		TRCN0000051526			
		TRCN0000290298			
MSN	3	TRCN0000333264			
		TRCN0000062408			
		TRCN0000062411			
PROM1	3	TRCN0000062145			
		TRCN0000062147			
		TRCN0000062144			
SERPINE2	3	TRCN0000290226			
		TRCN0000290224			
		TRCN0000052316			
TGFBI	3	TRCN0000291484			
		TRCN0000062173			
		TRCN0000062175			

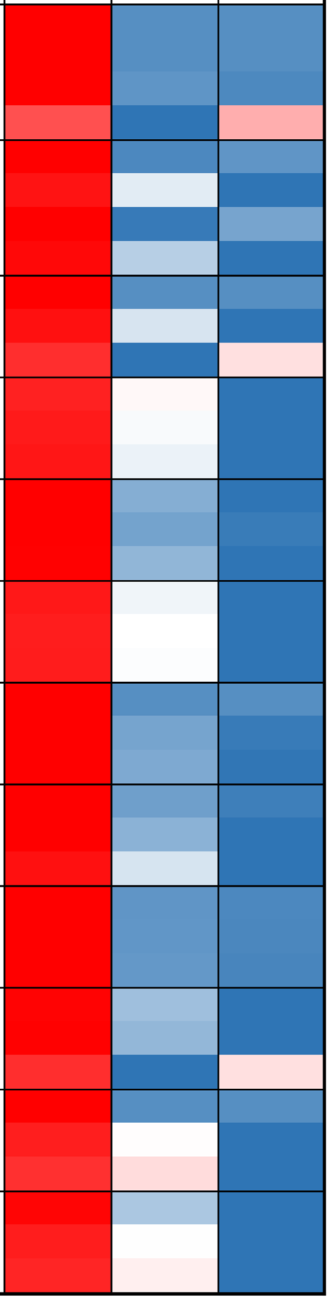

Supplementary Figure 3: A cluster diagram of genes with synthetic lethal interactions in regard to mutant *TP53* (R248Q). A total of 12 genes (with more than two numbers of shRNAs) were found to demonstrate synthetic lethality between LOF (loss-of-function) of p53 and shRNA lentiplex-depleted GOF (gain-of-function) of R248Q mutant p53 in basal-like TNBC cell lines carrying R248Q mutant TP53 (HCC70 and HCC1143), compared with BC cells with wild-type *TP53* (MCF-7). Red indicates higher than average expression and blue indicates lower than average expression.

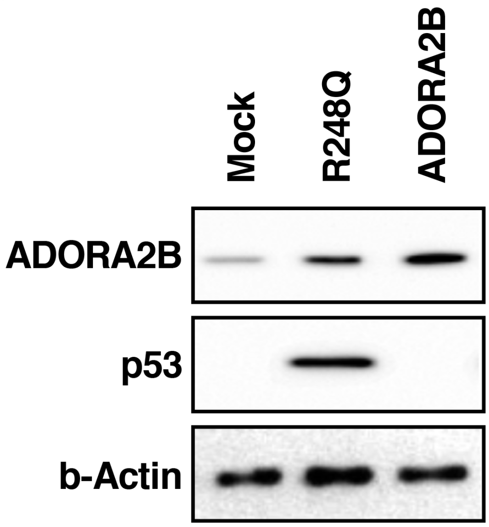

Supplementary Figure 4: Mutant *TP53* (R248Q) induces *ADORA2B* expression and tumorigenesis in breast cancer cells. (A) The breast cancer cell line HCC-70 was treated with p53 (R248Q) mutant and ADORA2B. Cell lysates were subsequently analyzed by immunoblotting analysis using anti-ADORA2B, TP53, or β-actin antibodies.
